# One of a Kind-The Pan African Clinical Trials Registry, a regional registry for Africa

**DOI:** 10.4314/pamj.v9i1.71221

**Published:** 2011-08-23

**Authors:** Amber L Abrams

**Affiliations:** 1 Amber L Abrams, South African Cochrane Centre, South African Medical Research Council

**Keywords:** Clinical Trial Registration, Registry, Primary Register Network, publication bias, transparency

## Abstract

The 2004 Ministerial Summit on Health Research called on the World Health Organization to to establish a registry network with the intention of providing a single access point to identify trials. In 2007 the International Committee of Medical Journal Editors amended their support of this initiative stating that only trials registered prospectively on a member registry of the WHO's Network of Primary Registers would be published. The Pan African Clinical Trials Registry (www.pactr.org), was established in early 2007 as the AIDS, TB and Malaria (ATM) Clinical Trials Registry with the aim of piloting the concept of a registry that would cater to the specific needs of African trialists. In 2009 the ATM Registry expanded its remit to include all diseases for all regions of Africa; The Pan African Clinical Trials Registry became the first and is presently the only African member of the World Health Organization's Network of Primary Registers.

## Background

Evidence from clinical trials can best answer research questions exploring which medicines or interventions work, and whether they cause harm [1]. Since clinical trials provide valuable information, clinicians and researchers should ideally have access to such information in order to inform practice. Until recently, very little attention was paid to collecting clinical trial data from the outset of a trial, and more often than not, information about trials reached clinicians and other researchers through journal articles and other publications. In the early 1990s there was increasing awareness on the issue of selective reporting and publication bias [2, 3].

Publication bias is the tendency for negative or neutral trial outcomes to be refused publication while positive and significant trial results are preferentially published [4]. This can lead to a skewed presentation of facts in the public record. To overcome this, prospective trial registration arose as a means to reduce the effects of publication bias and to encourage greater public disclosure [5]. Prospectively registered trials are housed in a clinical trial registry-a database in which important information (administrative and scientific) about planned, in-progress and completed trials, adequate to identify that trial's existence, are stored.

To this end, the Ministerial Summit on Health Research in 2004 called on the World Health Organization (WHO) to establish a registry network with the intention of providing a single access point to identify trials. This call was endorsed by 58th World Health Assembly (2005) to which the International Committee of Medical Journal Editors (ICMJE) released a statement of support. The ICMJE updated their statement in 2007, mandating that only trials prospectively registered on a member registry of the WHO's Network of Primary Registers would be published, making trial registration a minimum standard for publication in ICMJE member-journals.

## The Pan African Clinical Trials Registry

The Pan African Clinical Trials Registry (www.pactr.org), managed by the South African Cochrane Centre at the Medical Research Council, was established in early 2007 as the AIDS, TB and Malaria (ATM) Clinical Trials Registry. It aimed to pilot the concept of a registry that would cater to the specific needs of African trialists. Since there was no registry on the continent that met the requirements of the ICMJE or the WHO, in late 2008 the African Vaccine and Regulatory Forum (AVAREF) supported by the WHO-AFRO office recommended that ATM expand its remit beyond its disease-specific scope.

In June 2009, the Pan African Clinical Trials Registry was borne out of this expanded remit; the registry now accepts clinical trial applications pertaining to all diseases throughout Africa. In July 2009, www.pactr.org became the first and only member of the Network of WHO Primary Registers in Africa. Acknowledging that researchers who conduct clinical trials in Africa face challenges that others may not (for example, expensive or undependable internet access), www.pactr.org offers alternative modes of registration aside from the internet. Trials can be registered free of charge. The central goal of the registry is to collect prospective data on clinical trials in the region, and to display that data in the open-access repository online.

**Figure 1:**
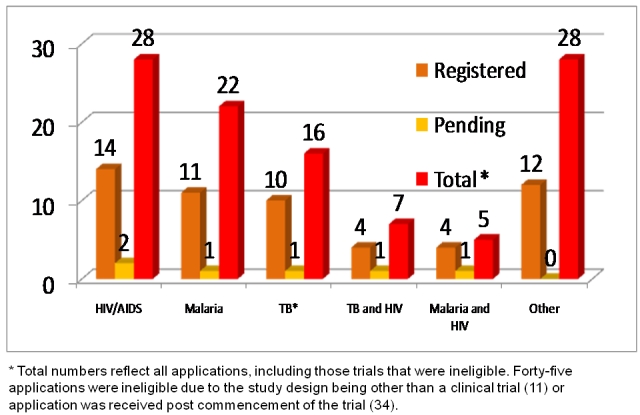
Total trial applications by disease type

**Figure 2:**
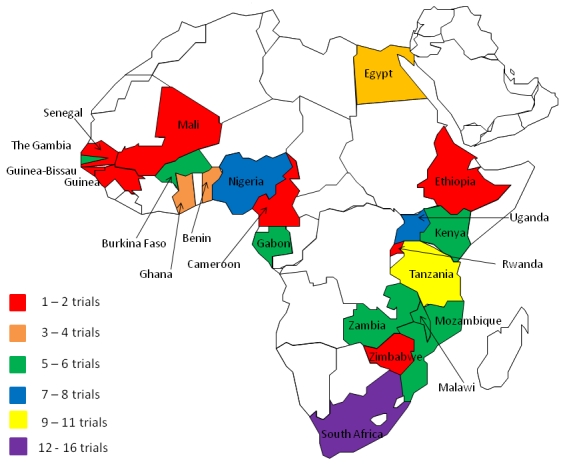
Locations of registered trial sites

In the past year, the registry has experienced substantial growth; applications to the registry have more than doubled. At present, www.pactr.org has received 106 applications and 55 trials have completed the registration process and been assigned registration numbers (Figure 1, Figure 2). Since one goal of the registry is to assist with minimising publication bias, at present www.pactr.org only accepts trials registered before first contact with patients (i.e., prospectively). Of the 106 trial application thirty-four were not registered prospectively which means that the trial protocol and methods cannot be tracked. Since trials that have not registered prospectively may have had the opportunity to change certain key aspects, like the intended outcomes of the trial, a registry must either notify users of late registration or exclude them from the public repository. At the moment our registry deals with trials of this nature in the latter way, but we are moving towards a system that would notify users in an effort to collect and publicly display as much trial data as possible.

One of the key objectives for www.pactr.org is to disseminate the registry data and the importance of clinical trial registration as a means of transparency, as widely as possible. To overcome the issue of limited internet access, www.pactr.org promoted their existence and presented their data at a number of international and local conferences and provided lectures to undergraduate and postgraduate students working in relevant fields.

Aside from its primary objective of registering clinical trials on the continent, www.pactr.org aims to increase transparency and self-sufficiency amongst national regulatory bodies to encourage clinical trial monitoring in Africa and world-wide. www.pactr.org has begun to work with national registries assisting with the establishment of their own local systems of oversight, registration and regulation. With first-hand experience in the development of our database in this resource constrained region, www.pactr.org was invited by the WHO and the Pan American Health Organization (PAHO) to facilitate working groups and share our experience (and challenges) with those aiming to develop a Latin American and Caribbean regional registry. In addition, in November 2010, www.pactr.org contributed to the first Working Group for the Pan African Clinical Trials Alliance (PACTA), a WHO supported initiative that brought together African national representatives with the aim of piloting aspects of registry and regulatory development. Promoting the registry and educating trialists on the importance of clinical trial registration has been a central focus of 2010.

### The Child Strategy – Clinical trials and children in Africa

www.pactr.org is also participating in a WHO initiative to develop an Africa-region Child Strategy with the intent of encouraging registration and conduct of clinical trials involving children. Through a grant administered by the World Health Organization (WHO) and funded by the Bill and Melinda Gates Foundation to support the development of regional Child Strategies in resource-constrained settings, staff of www.pactr.org developed the Africa-region Child Strategy. Since www.pactr.org aims to increase clinical trial registration in Africa by increasing awareness of the need to register trials and supporting trialists during registration, partnering with WHO to develop a child strategy falls within the structure and aims of www.pactr.org.

In December 2009, as a sub-project within the registry, the Child Strategy was launched. The strategy's objectives are: 1) To develop an awareness of the global and continental need to conduct and register clinical trials on paediatric trial research within the region; 2) To encourage trials in the region to include children as participants; and 3) To increase the number of registered trials including children in Africa.

The child strategy's dedicated Special Interest Group advises on the development of the research network and focused advocacy activities.

The Africa-region Child Strategy has made efforts to raise awareness of the need to conduct and register clinical trials with children as participants through the development of a database of interested stakeholders, through publications and presentations, and the mapping of past child-focused trial activity in the region. The child strategy began by developing a network of child-focused stakeholders including researchers, funders, institutions, non-profits, development agencies and policymakers, to increase collaboration and information sharing. Attendance at relevant conferences and agenda-setting meetings has heightened awareness, as have efforts to disseminate relevant research in areas relating to clinical trial support and trial environments. At present, 34% of registered trials on www.pactr.org include paediatric trials, with 48% of total registered trials dealing with issues that affect maternal and child health.

## Conclusion

The Pan African Clinical Trials Registry (www.pactr.org) is a regional clinical trials registry born out of the need to fill the gap in this form of oversight and as a means of preventing publication bias. The registry itself is growing at a rapid rate, as are activities relating to the goals of the registry. The Child Strategy, which aims to increase awareness of the need to conduct research in children, is a sub-project within the registry, and is also steadily picking up momentum. The last year of the project has focused on increasing awareness of the role of the registry, and the success of these efforts can be seen in the growth of the registry. www.pactr.org is a key resource for researchers, clinicians and policy makers on the continent.
